# Resource utilisation and costs of dementia in LMICs using patient‐level data: a systematic review

**DOI:** 10.1002/alz70861_108974

**Published:** 2025-12-23

**Authors:** Maria Kathia Cardenas, Christopher R. Butler, José Leal, Filipa Landeiro

**Affiliations:** ^1^ University of Oxford, Oxford, Oxfordshire UK; ^2^ Imperial College London, London UK; ^3^ The George Institute for Global Health, Sydney Australia

## Abstract

**Background:**

Dementia imposes a significant economic burden worldwide, particularly in low‐ and middle‐income countries (LMICs), where the majority of people with dementia (PWD) live. Understanding the costs of dementia and resource utilisation is essential to inform future cost‐effectiveness analyses and economic modelling. We aimed to describe and analyse resource utilisation and costs across all stages of dementia in LMICs.

**Method:**

We conducted a systematic review following a PROSPERO‐registered protocol (CRD42017071413). We identified studies using patient‐level data, published between 1 January 2000 and 14 August 2023, across several databases and registries. Data were selected, extracted, and quality appraised by two independent reviewers. The main outcomes were resource utilisation and cost per PWD. Outcomes were annualised and reported as total costs, by cost category and disease stage. Costs were adjusted for inflation and converted to international dollars using 2024 purchasing power parities (PPP). We assessed quality using a shortened version of the BMJ guidelines.

**Result:**

The review included 23 studies, of which 21 provided unique patient‐level datasets, covering 14 countries and 14,392 PWD. Resource utilisation was not reported separately in most studies, and results varied widely: inpatient admissions ranged from 0 to 4.9 per year, outpatient visits from 0.4 to 14.9 per year, and informal caregiver time from 0.2 to 17.9 hours/day. Only 43% reported the mean annual total cost per PWD by disease stage. Mean annual total cost per PWD, irrespective of dementia stage, ranged from 4,658 to 37,887 USD PPP. Greater variation was observed across stages: from 1,316 to 27,438 USD PPP in mild dementia; 3,981 to 36,637 USD PPP in moderate; and from 7,520 to 51,456 USD PPP in severe dementia. Costs in severe dementia often doubled those in earlier stages. Few studies reported caregivers’ productivity losses, and none included pre‐dementia or end‐of‐life stages.

**Conclusion:**

Resource utilisation and costs of dementia increased substantially with disease progression, but the reporting of both outcomes was highly heterogeneous. There is an urgent need to improve the quality of reporting in studies from LMICs. Further research using patient‐level data is needed, given that only 10% of LMICs had at least one study.

